# Supplementary material for: Chromatin structure and var2csa – a tango in regulation of var gene expression in the human malaria parasite, Plasmodium falciparum?
Source: bioRxiv. 2024 Feb 13:2024.02.13.580059. Preprint. [Version 1] doi: 10.1101/2024.02.13.580059 (PMC10888805; doi:10.1101/2024.02.13.580059)
Supplement: Supplement 1 — Figure S1. Differential expression analysis. (A) Spearman correlation for genome-wide expression in both replicates and cell lines. (B) Differential expression for the top 300 most variable genes among all samples with the var genes highlighted in red on the left side of the heatmap. (C) GO enrichment analysis for up- and downregulated genes in the differential expression dataset. Figure S2. Differential peak calling between the WT and ∆V2 cell lines. (A) Pearson correlation of H3K9me3 peaks between all samples. (B) Number of unique and overlapping significant peaks for each cell line. (C) Deviation from mean read count in significant differential H3K9me3 binding sites. Differential peaks are mapped to genes within 1 kb of coding region. Figure S3. Hi-C correlation analysis. (A) Stratum-adjusted correlation between all samples used in the Hi-C analysis. (B) Negative log-linear relationship between genomic distance and contact probability. Figure S4-S5. Hi-C interaction heatmaps for the WT (Fig. S4) and ∆V2 (Fig. S5) cell lines. Intrachromosomal interaction heatmaps (A-N) for each chromosome and an interchromosomal interaction heatmap (O) all binned at 10 kb resolution. All data is ICED normalized and WT sample is subsampled to the same read depth as ∆V2 with the color scaled to the highest value for each chromosome between both datasets. Var genes are indicated along the top and right side of each heatmap in red and the centromeres are shown in gray. Figure S6. Differential Hi-C contact heatmaps. (A) Differential intra- and interchromosomal interaction heatmaps identifying regions with a positive (red) and negative (blue) log2 fold change in ∆V2 over WT. Bins containing var genes are indicated in red and centromeres in gray along the top and side of each heatmap. Figure S7. Var gene differential interactions and compartment analysis of the WT. (A) Differential interactions for 10 kb bins containing var genes. Var genes that fall within two bins are mapped to the bin [file NIHPP2024.02.13.580059v1-supplement-1.pdf]

Figure S2

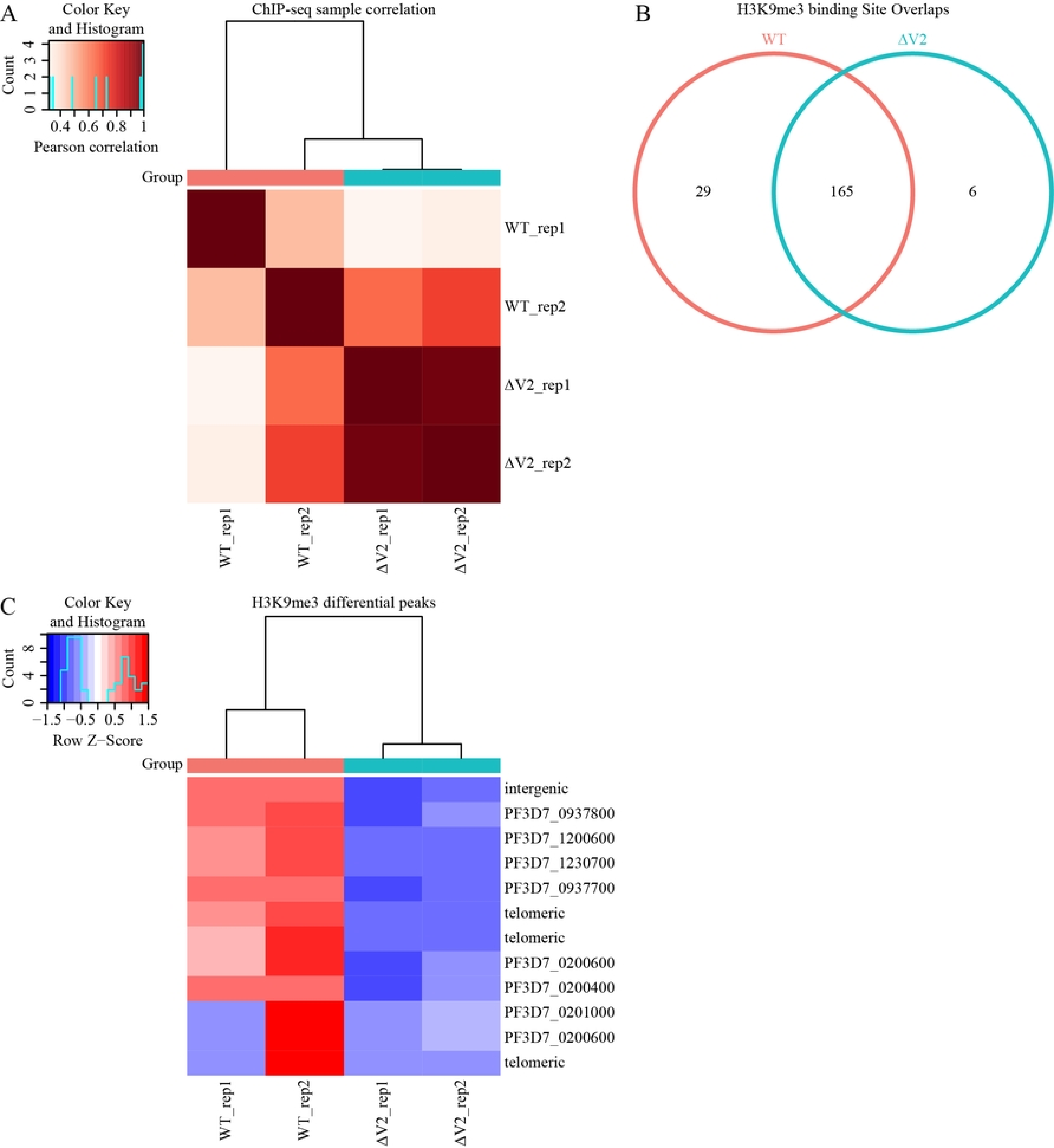

Figure S2

Figure S3

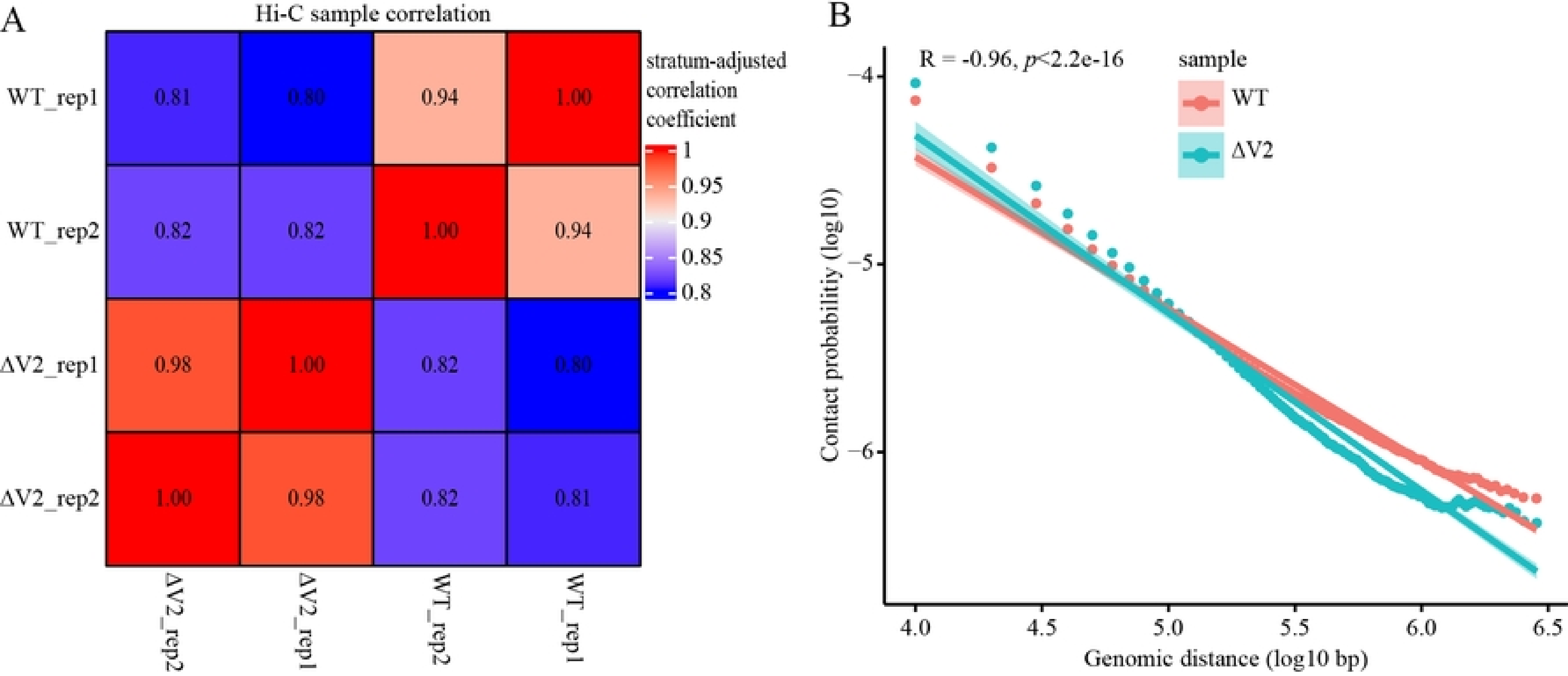

Figure S3

Figure S4

WT chromatin interactions

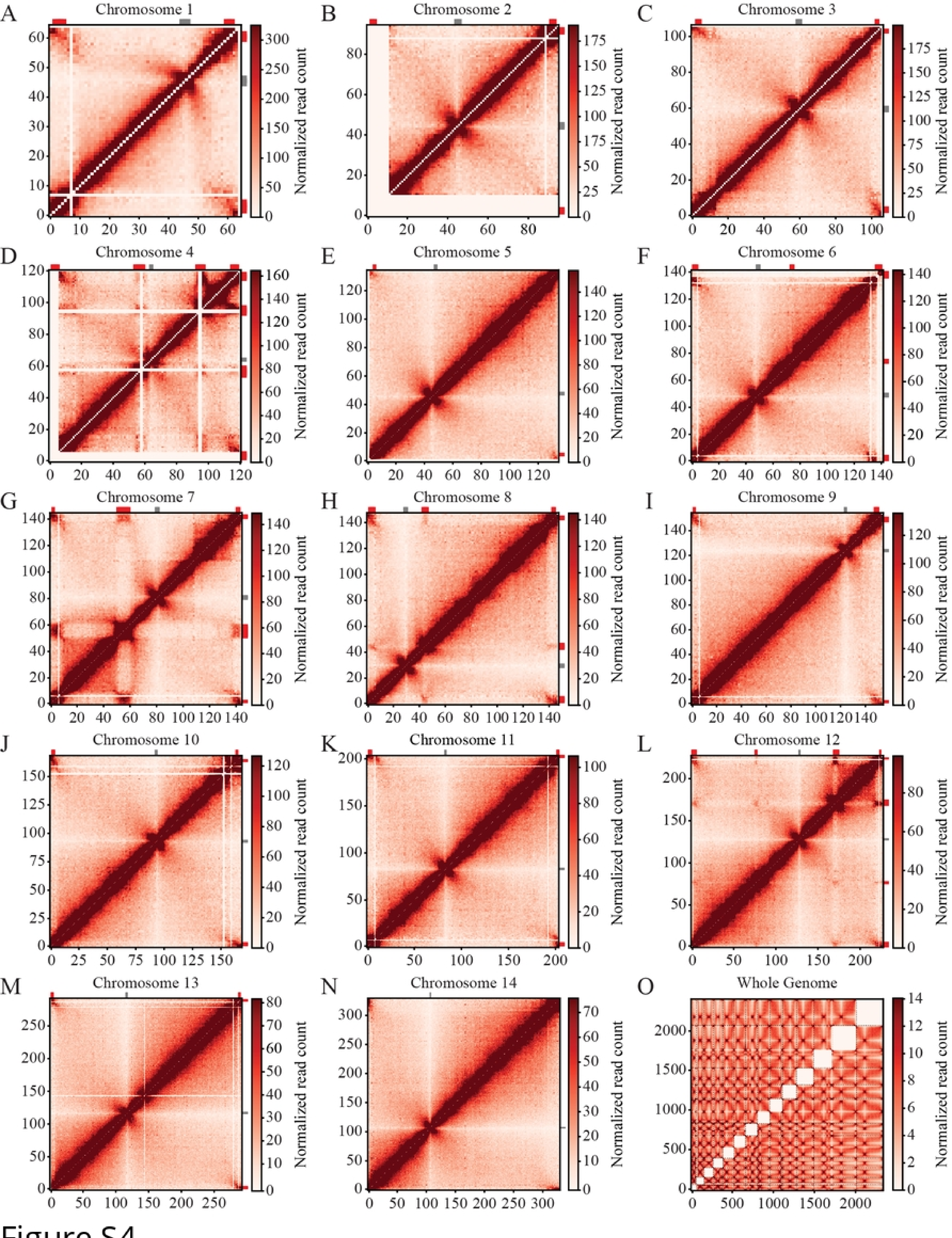

Figure S4

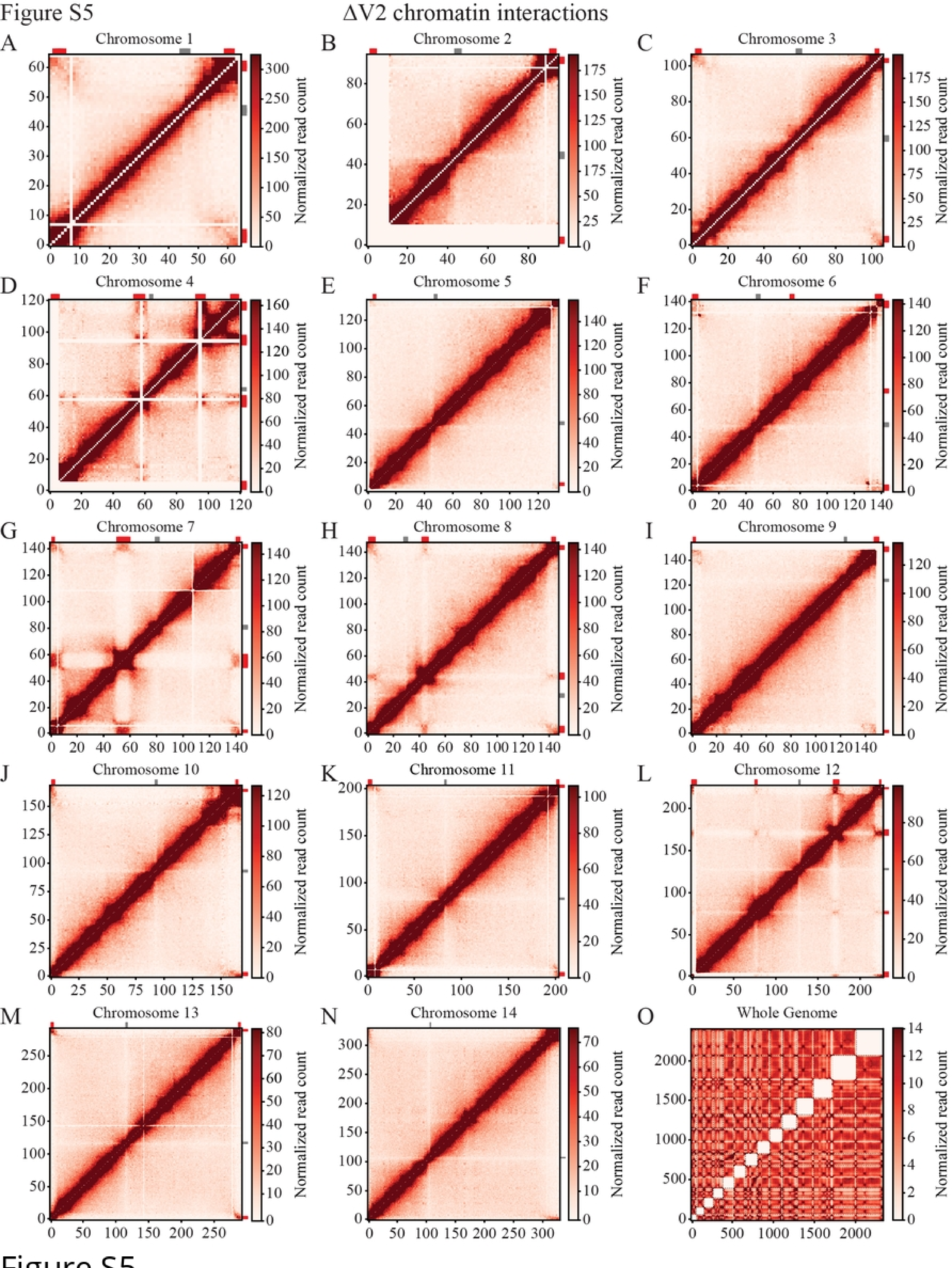

Figure S5
ΔV2 chromatin interactions
A
Chromosome 1
B
Chromosome 2
C
Chromosome 3
D
Chromosome 4
E
Chromosome 5
F
Chromosome 6
G
Chromosome 7
H
Chromosome 8
I
Chromosome 9
J
Chromosome 10
K
Chromosome 11
L
Chromosome 12
M
Chromosome 13
N
Chromosome 14
O
Whole Genome
Normalized read count

Figure S5

Figure S6 Differential chromatin interactions

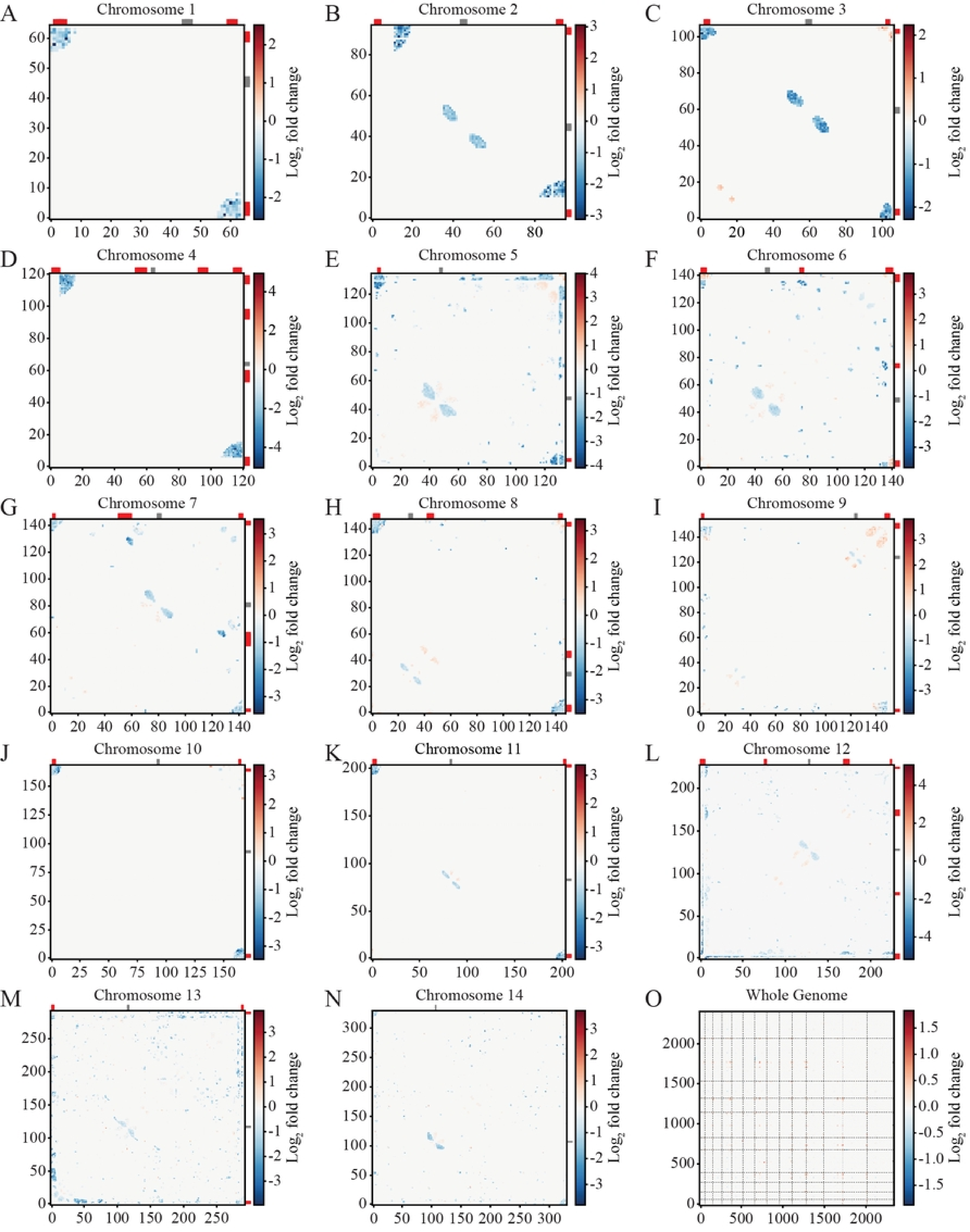

Figure S6

Figure S7

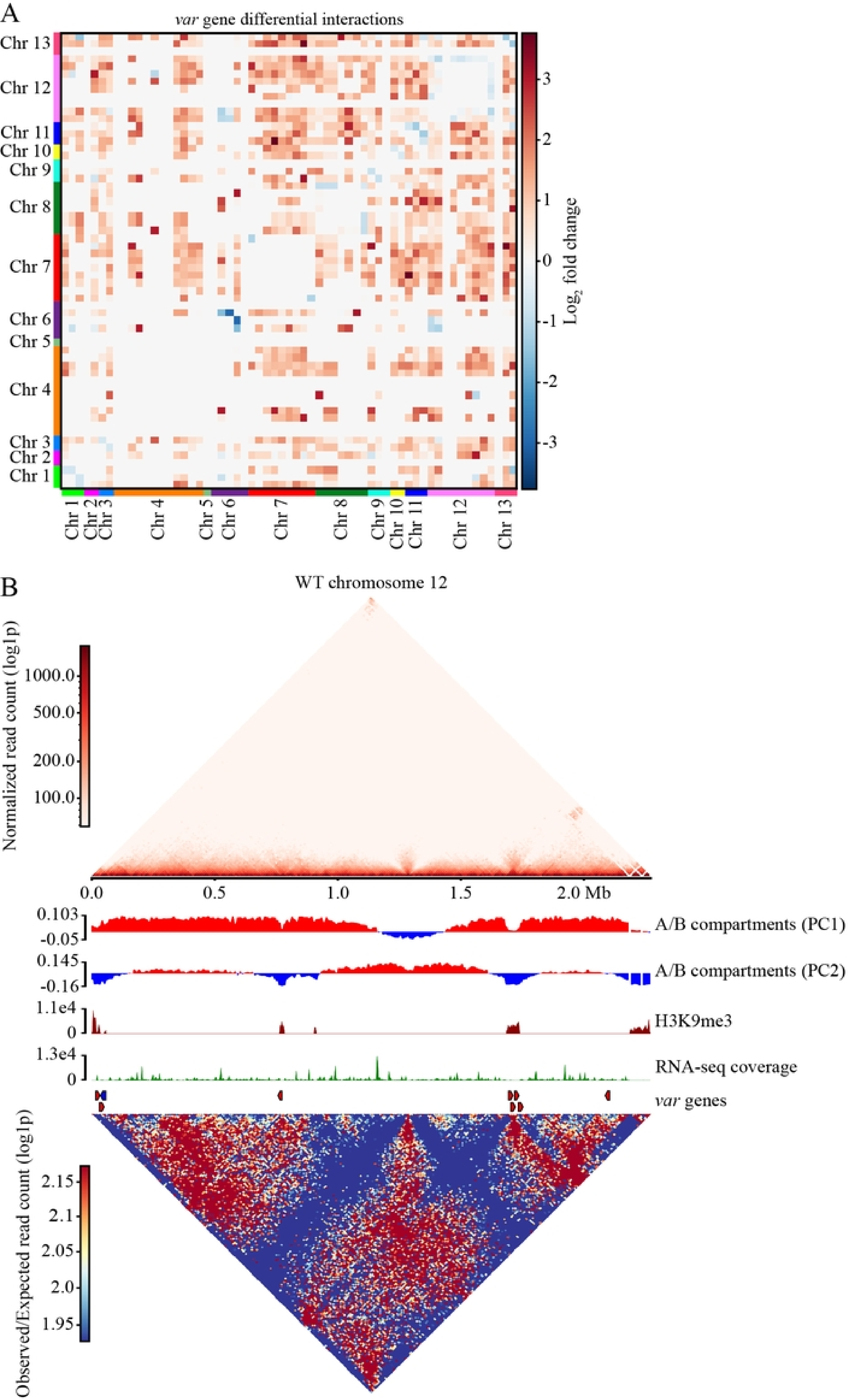

Figure S7

Figure S1

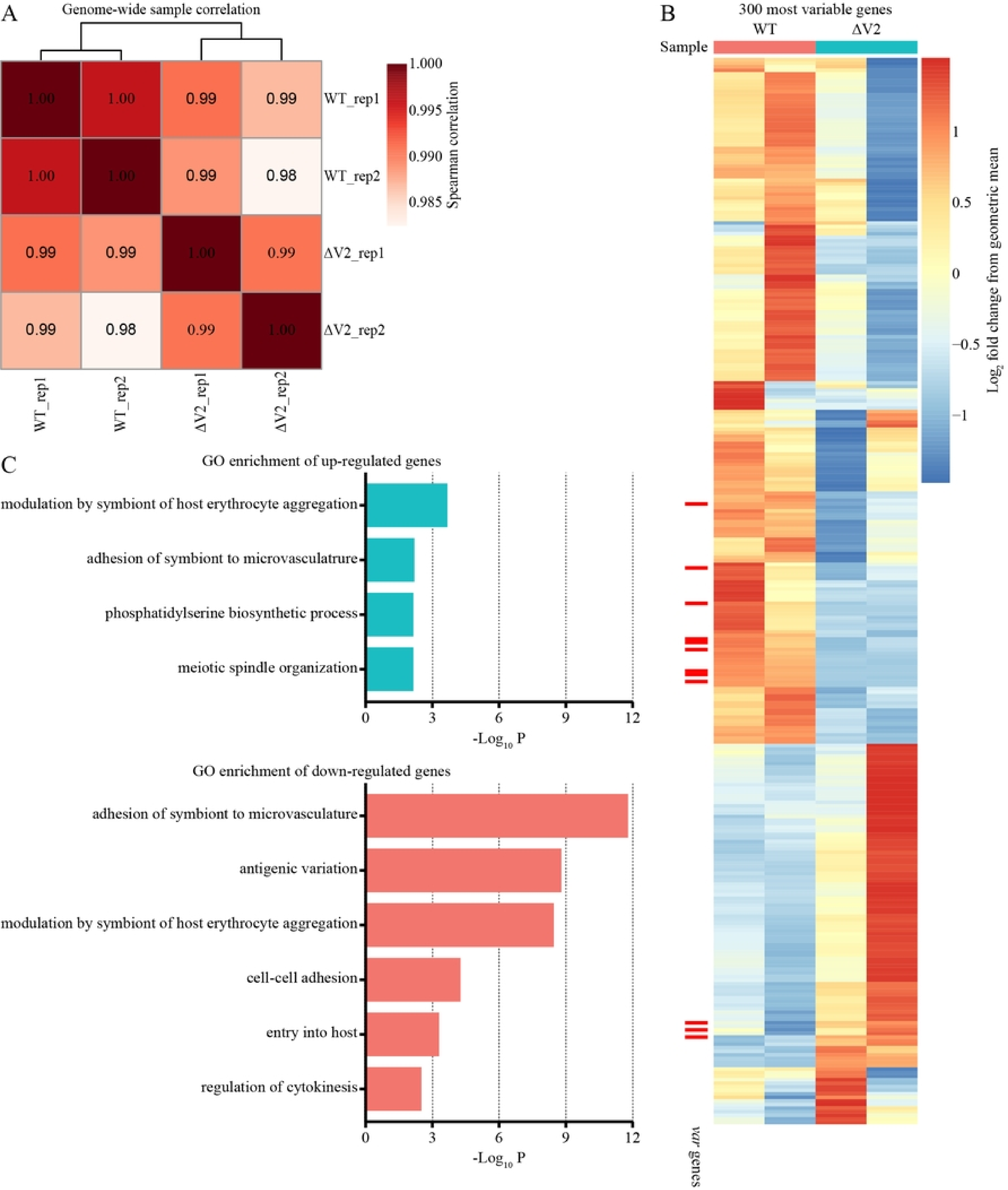

Figure S1
